# Integrating bibliometrics and real-world data to map varicocele research and predict surgical outcomes

**DOI:** 10.3389/fendo.2026.1837765

**Published:** 2026-04-23

**Authors:** Yu Zhou, Shenghui Zhu, Huimin Liang, Huan Li, Ruishan Wu

**Affiliations:** 1 Department of Andrology, National Health Commission (NHC) Key Laboratory of Male Reproduction and Genetics, Guangdong Provincial Reproductive Science Institute (Guangdong Provincial Fertility Hospital), Guangzhou, China; 2 Department of Laboratory Medicine, National Health Commission (NHC) Key Laboratory of Male Reproduction and Genetics, Guangdong Provincial Reproductive Science Institute (Guangdong Provincial Fertility Hospital), Guangzhou, China; 3 Assisted Reproductive Technology Center, Foshan Maternal and Child Health Care Hospital, Foshan, China

**Keywords:** bibliometrics, male infertility, microsurgical varicocelectomy, semen parameters, varicocele

## Abstract

**Background:**

Varicocele represents the most prevalent amendable factor contributing to male infertility. Despite this, considerable debate continues concerning the effectiveness of surgical intervention, especially in terms of enhancing pregnancy outcomes. Simultaneously, the body of literature in this domain is expanding swiftly, necessitating systematic organization.

**Objective:**

This study seeks to delineate the research landscape surrounding varicocele, evaluate the effectiveness of surgical treatment, and investigate critical predictive factors affecting surgical outcomes by synthesizing bibliometric analysis with empirical clinical data.

**Methods:**

Initially, we extracted pertinent literature spanning from 2004 to 2025 from the Web of Science Core Collection. Utilizing bibliometric analysis tools such as CiteSpace, VOSviewer, and Bibliometrix R, we analyzed 2,186 publications to assess research trends, contributions by country, prominent authors, journal distribution, and emerging research hotspots. Subsequently, we conducted an empirical study involving 113 infertile patients who underwent microsurgical varicocelectomy (MSV). We compared their semen parameters before and after the surgery and employed multiple linear regression and random forest models to evaluate the influence of factors such as age, venous diameter, and reflux duration on surgical outcomes.

**Results:**

The bibliometric analysis demonstrated a consistent annual growth in publications within this field. The United States, China, and Italy emerged as the leading contributors. Agarwal A was identified as the most prolific and influential author. Current research frontiers are centered on oxidative stress and sperm DNA fragmentation. A real-world study substantiated that MSV significantly enhanced various semen parameters. Specifically, sperm concentration, total sperm count, the percentage of progressively motile sperm, and the total number of progressively motile sperm increased by 68.1%, 84.2%, 53.7%, and 135.7%, respectively. Predictive modeling revealed that reflux duration, preoperative sperm concentration, and age were the most critical factors affecting the improvement in the total number of progressively motile sperm. Conversely, venous diameter was identified as the primary factor influencing enhancements in the percentage of progressively motile sperm and the sperm abnormality rate.

**Conclusion:**

Our findings confirm that microsurgical repair significantly improves semen parameters. We therefore advocate for a refined varicocele classification system that incorporates venous diameter, patient age, and reflux duration to enhance clinical decision-making and patient selection.

## 1 Introduction

Varicocele, defined by the abnormal dilation and tortuosity of the pampiniform plexus within the scrotum, is the most prevalent correctable cause of male infertility, impacting approximately 15-20% of the general male population and 35-40% of men with primary infertility (1, 2).

The pathophysiological mechanisms linking varicocele to impaired spermatogenesis are multifaceted, involving factors such as testicular hyperthermia, venous pressure-induced hypoxia, oxidative stress, and the accumulation of reactive oxygen species (3). These factors collectively disrupt the spermatogenic microenvironment, resulting in reduced sperm count, motility, and morphology (4–6).

Despite decades of extensive research and the well-documented association between varicocele and abnormal semen parameters, significant controversies remain regarding the universal efficacy of varicocele repair (5, 7). The primary debates focus on the extent to which surgical intervention enhances semen quality and, more importantly, whether these improvements consistently lead to increased natural pregnancy rates and live births (8, 9). Consequently, discussions continue within clinical guidelines concerning optimal patient selection and treatment strategies. Concurrently, the scientific literature on varicocele has expanded rapidly, resulting in a vast and complex body of knowledge that is challenging to navigate using traditional review methods. Bibliometrics, a cross-disciplinary science that utilizes quantitative analysis and statistics to elucidate publication patterns, offers a powerful tool for mapping this evolving field (10–12).

The study employs a dual-method approach to address the limitations of bibliometric analysis in resolving clinical controversies. While bibliometric analysis effectively identifies research trends, core topics, influential studies, and collaborative networks, providing a macroscopic overview of scientific discourse, it is insufficient for elucidating the underlying reasons for observed outcomes or optimizing clinical decision-making. To bridge this gap, we first conduct a comprehensive bibliometric analysis of global varicocele literature from the past two decades, aiming to delineate the research landscape, its evolution, and current frontiers. Subsequently, we integrate these insights with a real-world clinical study that examines surgical outcomes to empirically investigate predictors of treatment success. The ultimate objective of this integrated analysis is to synthesize evidence from published literature with original clinical data, thereby contributing to the development of more precise, evidence-based criteria for varicocele management.

The rationale for integrating bibliometric analysis with clinical data is twofold. First, bibliometric mapping reveals the intellectual structure of varicocele research—identifying prevailing topics (e.g., oxidative stress, sperm DNA fragmentation), influential studies, and persistent controversies (e.g., whether surgical benefits translate into pregnancy outcomes). Second, these identified knowledge gaps directly inform our clinical investigation: we focus on predictors of postoperative semen improvement—specifically age, reflux duration, and venous diameter—which emerged as underexplored yet potentially critical factors in the bibliometric analysis. Thus, the bibliometric component serves as a hypothesis-generating tool, while the clinical component provides hypothesis-testing validation within a real-world cohort.

## 2 Method

### 2.1 Literature retrieval

**Figure 1:**
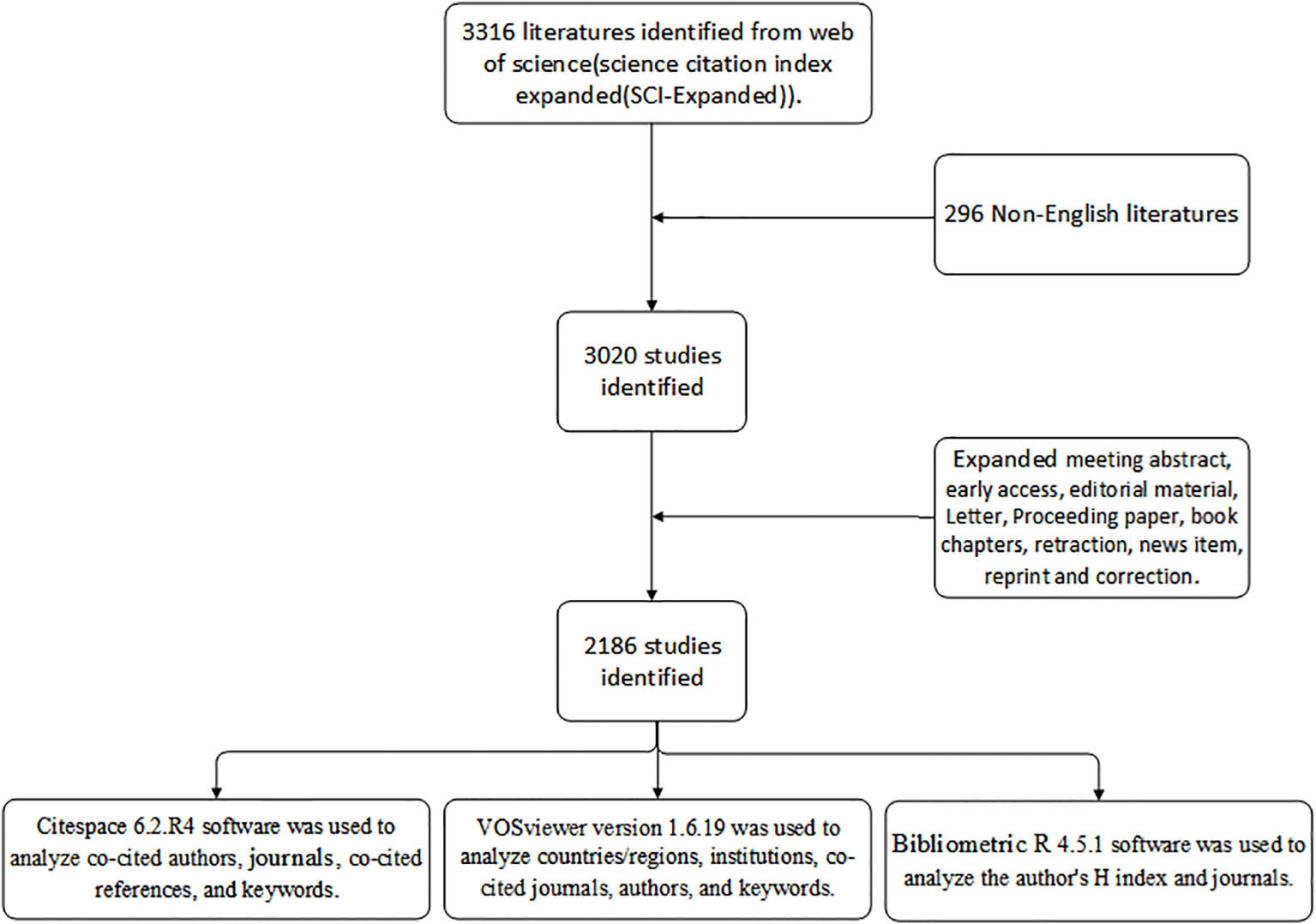
The flowchart for searching and analyzing the varicocele.

Bibliometric data on varicocele were retrieved from the Science Citation Index Expanded (SCI-EXPANDED) within the Web of Science Core Collection (WoSCC), a widely recognized repository for quantitative science mapping (13–15) (**Figure 1**). The search was conducted on August 25, 2025, covering literature published between January 1, 2004, and June 30, 2025. Using the query “varicocele OR varicoceles,” we searched the title, abstract, and author keyword fields. Following automated deduplication and relevance screening, only English-language articles and reviews were retained. Full records, including titles, authors, abstracts, keywords, and references, were exported as plain text files for subsequent analysis.

### 2.1.1 Visual analysis

Using Citespace software (version 6.2.R4), VOSviewer software (version 1.6.19), Biblimetix R (version 4.5.1), and Microsoft Office Excel 2021 (Microsoft, Redmond, Washington, USA), we conducted an extensive visual analysis of the literature about meeting requirements. These software tools were used to analyze publications, authors, countries/regions, journals, references, and keywords in the varicocele literature.

### 2.2 Real-world research

#### 2.2.1 Patient data

After strict screening of men diagnosed with infertility and varicocele after visiting Guangdong Provincial Reproductive Hospital (Guangdong Provincial Institute of Reproductive Sciences) from January 2023 to May 2025, we finally enrolled 113 infertile patients with varicocele hospitalized. All patients underwent surgery after signing an informed consent form. The age of onset, course of disease, and physical examinations, scrotal color Doppler ultrasound before surgery, and semen examinations before and after surgery were performed. This study was approved by the Ethics Committee of Guangdong Provincial Reproductive Science Institute (Guangdong Provincial Fertility Hospital) (Approval No. 2026 (04)).

#### 2.2.2 Inclusion criteria

Participants were eligible for inclusion if they satisfied all of the following criteria: 1) A diagnosis of primary or secondary infertility, characterized by the inability to achieve pregnancy following a minimum of 12 months of unprotected sexual intercourse. 2) The presence of a clinically palpable varicocele, graded I to III, as confirmed by scrotal color Doppler ultrasound (CDU). 3) At least one abnormal semen parameter as defined by the World Health Organization’s laboratory manual (5th edition), such as a sperm concentration of less than 15 million/mL, progressive motility below 32%, or normal morphology under 4%. 4) Completion of a microsurgical subinguinal varicocelectomy (MSV).

#### 2.2.3 Exclusion criteria

Patients were excluded from the study if they met any of the following criteria: 1) Azoospermia, whether obstructive or non-obstructive; 2) Presence of genetic abnormalities, such as Klinefelter syndrome or Y-chromosome microdeletions; 3) A history of acute genital tract infection within the preceding three months; 4) Concurrent presence of other conditions known to severely affect fertility, including bilateral cryptorchidism or pituitary dysfunction; 5) A female partner diagnosed with significant infertility factors, such as bilateral tubal obstruction, severe endometriosis, or anovulation.

#### 2.2.4 Sperm analysis

In accordance with the World Health Organization’s “Laboratory Manual for the Examination and Processing of Human Semen” (5th Edition, 2010), semen samples were obtained through masturbation in a sterile environment following a period of abstinence ranging from 2 to 7 days. The collected samples were liquefied in a 37 °C water bath and subsequently analyzed using the computer-assisted sperm analysis (CASA) system (Microptic SL, Barcelona, Spain). Sperm volume was determined using the Weighing method, while CASA was employed to evaluate additional parameters. Each sample underwent immediate analysis in a counting chamber, where at least 200 sperm were assessed across five microscopic fields. The recorded parameters included total sperm count, concentration, motility, and progressive motility. Sperm exhibiting a path velocity exceeding 5 mm/s were classified as motile, whereas those with a velocity greater than 25 mm/s and linearity above 80% were designated as “progressively motile.” These parameters were aligned with the standards outlined in the World Health Organization Laboratory Manual for the Examination and Processing of Human Semen (Fifth Edition) (16), which specifies sperm volume at 1.5 ml, sperm count at 39 million, sperm concentration at 15 million/ml, total motility at 40%, and progressive motility at 32%. Sperm morphology was analyzed using Papanicolaou staining.

#### 2.2.5 Scrotal ultrasound examination

In a private clinical setting maintained at a temperature of 26 °C, a Mindray R8 color Doppler ultrasound equipped with a 7.5–12 MHz linear probe was utilized. The patient was positioned supine, with the lower abdomen and external genitalia exposed, and the penis secured to the abdominal wall. The ultrasound probe was strategically placed above the testis to acquire sagittal images, facilitating the measurement of the maximum inner diameter of the spermatic vein and the width of the spermatic cord bilaterally. Additionally, the Valsalva maneuver spectrum and the reflux time of the spermatic vein were documented.

#### 2.2.6 Microscopic varicocelectomy

The patient is positioned supine, and general anesthesia is administered via endotracheal intubation. Following the induction of anesthesia, standard disinfection and draping procedures are conducted. A 2 cm incision is then made at the external ring. The spermatic cord is identified, mobilized, and exteriorized. Under microscopic guidance, all varicose veins are meticulously isolated and ligated, with careful attention given to preserving the arteries and lymphatic vessels. The surgical field is inspected for any missed ligations or bleeding. Subsequently, the spermatic cord is repositioned, and the incisions are closed sequentially.

### 2.3 Statistical analysis

#### 2.3.1 Statistical analysis and sensitivity analysis

Data analysis was performed using R software (version 4.5.1). Continuous variables were assessed for normality using the Shapiro-Wilk test. Since most parameters (e.g., DNA fragmentation index, sperm concentration) deviated significantly from a normal distribution (all P < 0.05), non-parametric tests were employed for inferential analysis. The Wilcoxon signed-rank test was used for paired comparisons (e.g., pre- vs. post-operative measurements), and the Kruskal-Wallis test was applied for comparisons across multiple groups. The Variance Inflation Factor (VIF) was calculated for all independent variables considered in the analysis, with values below 5 indicating the absence of severe multicollinearity.

To ensure the robustness of the primary findings, a sensitivity analysis was conducted. Data points where the improvement rate exceeded the upper whisker (defined as Q3 + 1.5 × Interquartile Range) were excluded. The results of this analysis confirmed that the main conclusions were not materially affected by these potential outliers.

#### 2.3.2 Variable categorization

To facilitate clinical interpretation, key continuous variables were categorized as follows:

To evaluate age-related effects on treatment outcomes, patients were stratified into three age groups (17): Young (≤30 years), Middle-aged (>30 and ≤40 years), and Older (>40 years).

Varicocele can be categorized into clinical and subclinical types based on clinical assessment and ultrasound diagnostics. The clinical type is further stratified into three grades (18):

1. Subclinical varicocele is characterized by the absence of palpable abnormalities, yet ultrasound evaluation during quiet breathing reveals a diameter range (DR) of 1.8-2.1 mm. There is no reflux observed under normal conditions, but reflux is present during the Valsalva maneuver, with a total reflux time (TR) of 1–2 seconds.
2. Clinical varicocele Grade I is identified by a positive palpation finding and an ultrasound DR of 2.2-2.7 mm during quiet breathing. Reflux is noted during the Valsalva maneuver, with a TR of 2–4 seconds.
3. Clinical varicocele Grade II presents with positive palpation and an ultrasound DR of 2.8–3.1 mm during quiet breathing. Reflux occurs during the Valsalva maneuver, with a TR of 4–6 seconds.
4. Clinical varicocele Grade III is distinguished by positive palpation and an ultrasound DR of 3.1 mm or greater during quiet breathing. Reflux is observed during the Valsalva maneuver, with a TR of 6 seconds or more.

Preoperative sperm concentration was categorized based on the World Health Organization (WHO) guidelines (15) into the following groups: severe oligozoospermia (<5 million/mL), moderate oligozoospermia (≥5 and <10 million/mL), mild oligozoospermia (≥10 and <15 million/mL), and normal (≥15 million/mL).

## 3 Results

### 3.1 Bibliometric analysis and visualization

#### 3.1.1 Publication and author analysis

**Figure 2:**
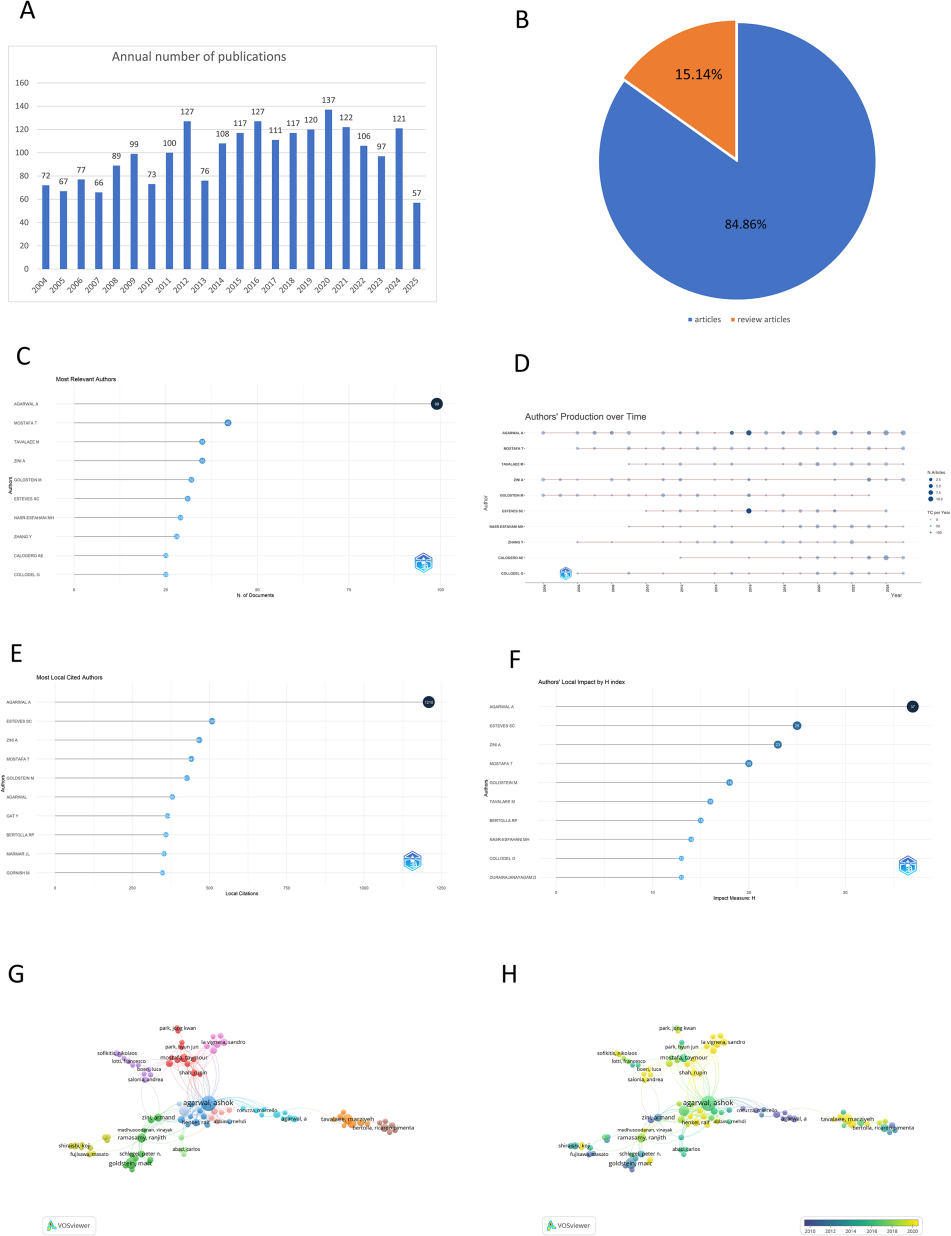
The number of publications and authors on varicocele. **(A)** The number of publications in every year. Only the number of articles published in the first eight months in 2025. **(B)** The distribution of included literature types. **(C)** The top 10 authors. **(D)** authors’ production over time. **(E)** The cited authors. **(F)** The top ten authors’ local impact H index. **(G)** The co-authorship authors network visualization. **(H)** The co-authorship authors overlay visualization. The thicker the line the stronger cooperation.

A total of 2186 publications met the search criteria (**Figure 2A**), comprising 1855 articles (84.86%) and 331 reviews (15.14%) (**Figure 2B**). Publications have steadily increased from 2004 to 2020, peaking in 2020, and have remained high over the past five years. Vosviewer analysis identified over 9202 authors in the varicocele field. **Figure 2C** highlights the top 10 authors, their productivity over time (**Figure 2D**), citations (**Figure 2E**), and H-index (**Figure 2F**). Agarwal A leads with 99 publications and 1210 citations, followed by Mostafa T, Tavalaee M, Zini A, and Goldstein M. Agarwal A also has the highest H-index at 37, with Esteves CS, Zini A, Mostafa T, and Goldstein M trailing. Author collaborations, mainly from 2010 to 2020, are visualized in **Figures 2G, H**.

#### 3.1.2 Countries/regions, institutions, and journals analysis

**Figure 3:**
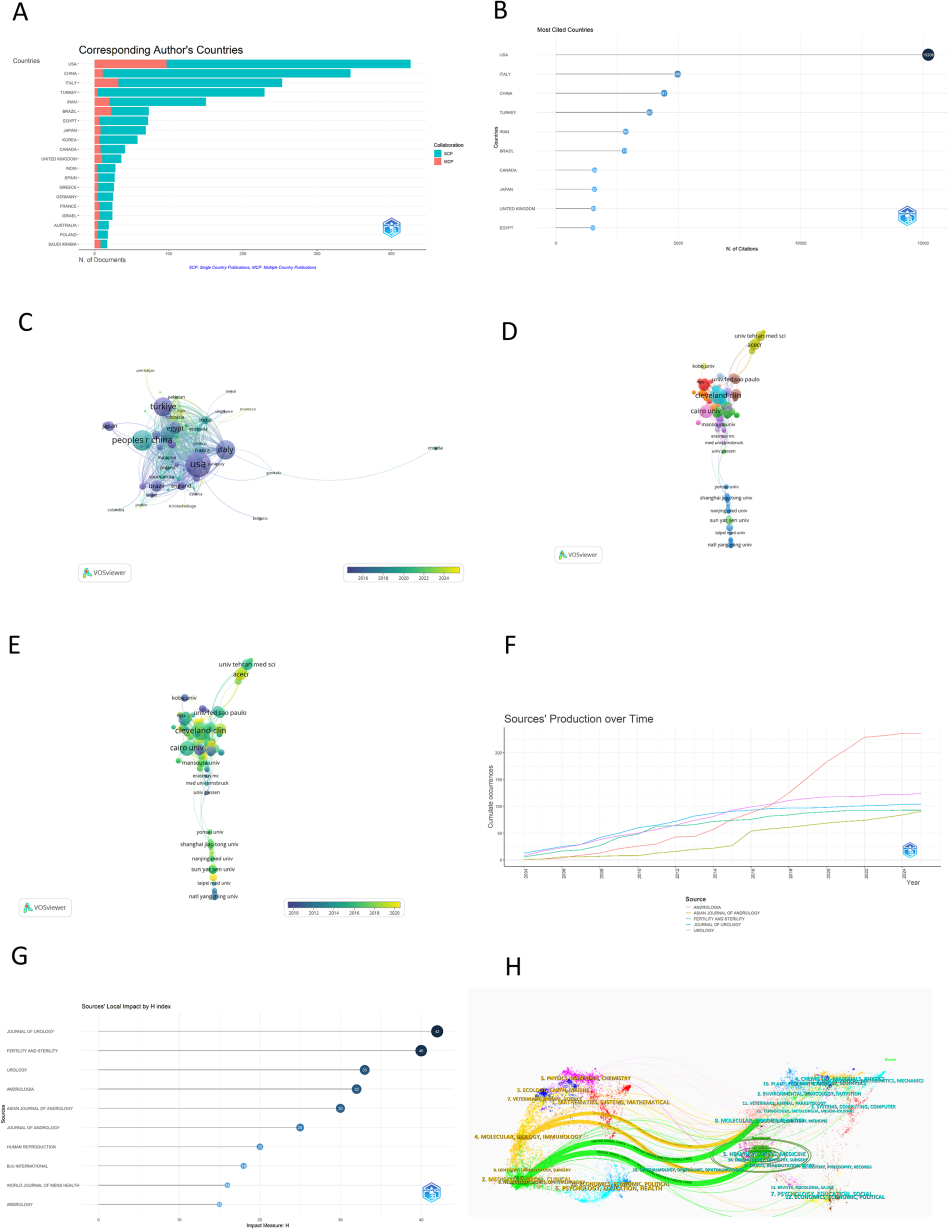
The published Countries/regionals, institutions and journals on varicocele. **(A)** The corresponding author’s countries. **(B)** The most cited countries. **(C)** The co-authorship countries/regionals overlay visualization. **(D)** The co-authorship institutions network visualization. **(E)**The co-authorship institutions overlay visualization. **(F)** The top five frequency journals’ production over time. **(G)** The journals local impact H index. **(H)** The journal dual-map.

A total of 90 countries and regions have contributed to research in the field of varicocele. Among the top ten contributors, the United States has produced the highest number of articles (426), followed by China (345) and Italy (253) (**Figure 3A**). Additionally, the USA is the most frequently cited country, with a citation count of 15,208 (**Figure 3B**). The United States also demonstrates significant collaboration with other countries and regions, particularly between 2010 and 2020 (**Figure 3C**). The top ten institutions with frequent publications in this domain are listed in **Table 1**, with the Cleveland Clinic leading by publishing 52 articles on varicocele. The collaborative networks among these institutions are illustrated in **Figure 3D**, with notable cooperative activities occurring from 2010 to 2020 (**Figure 3E**). The Biblimetix R analysis identifies 423 journals publishing on varicocele from 2004 to 2025, with the top five listed in **Table 2**. *Andrologia* is the most prolific, followed by *Urology* and *The Journal of Urology*. The impact factors of these top journals range from 2.0 to 7.5. **Figure 3F** shows *Andrologia*’s sharp production increase from 2018 to 2022. **Figure 3G** highlights *The Journal of Urology*’s top local H index of 42 among the top ten journals. **Figure 3H** presents a dual-map overlay of citation links between journals and their co-cited counterparts in the varicocele field.

**Table 1:** The top 10 frequency publishment institutions.

	Institution	Article	Citation
1	Cleveland Clinic	52	4273
2	Cairo University	39	1046
3	acecr	24	373
4	University of Verona	23	523
5	McGill University	22	1292
6	University of Toronto	21	861
7	University fed sao paulo	21	765
8	Tehran University of Medical Sciences	21	313
9	University of Siena	20	364
10	University of Sao Paulo	19	647

**Table 2:** The top five publishment journals.

Rank	Sources	Articles	Import factor (2024)		Country
1	Andrologia	236	2.0	Q3	United Kingdom
2	Urology	124	2.1	Q2	USA
3	Journal of urology	104	6.1	Q1	USA
4	Fertility and sterility	93	7.0	Q1	USA
5	Asian journal of andrology	91	2.7	Q2	China

#### 3.1.3 References and keywords analysis

**Figure 4:**
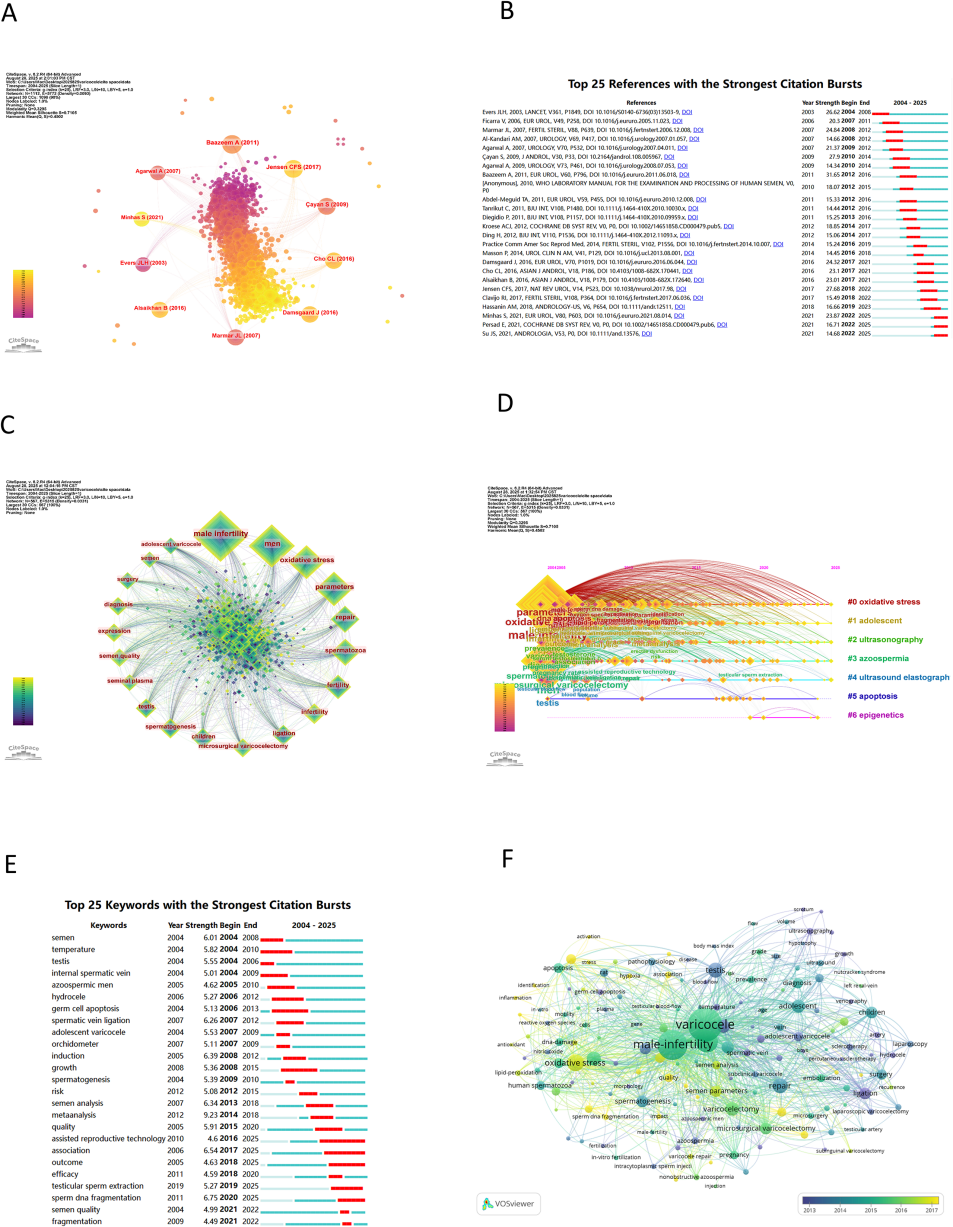
The References and keywords on varicocele. **(A)** The top ten co-cited references. **(B)** The top 25 references with the strongest citation bursts. **(C)** The top 20 keywords. **(D)** The clusters of keywords. **(E)** The top 25 keywords with the strongest citation bursts. **(F)** The keywords overlay visualization.

The top ten co-cited references and the top 25 references with the strongest citation bursts are shown in **Figures 4A, B**. Notably, the article titled “Varicocele and Male Factor Infertility Treatment: A New Meta-analysis and Review of the Role of Varicocele Repair” exhibits the highest citation burst score of 31.65. This study highlights the benefits of varicocelectomy in enhancing sperm parameters, such as count and motility, reducing sperm DNA damage and oxidative stress, and improving sperm ultramorphology. While various repair methods are effective, microsurgical repair is associated with superior outcomes (19). Another article “Treatment of Palpable Varicocele in Infertile Men: A Meta-analysis to Define the Best Technique” has a high citation burst of 27.9, concluding that MSV leads to better pregnancy rates and fewer complications than conventional methods. However, more extensive studies are needed to compare its efficacy with other treatments (20).

There are 20 keywords with over 100 occurrences related to varicocele, with the top 10 being: male infertility, men, oxidative stress, parameters, spermatozoa, repair, fertility, infertility, ligation, and microsurgical varicocelectomy (**Figure 4C**). According to the Citespace timeline view (**Figure 4D**), the top 7 keyword clusters are: #0 oxidative stress, #1 adolescent, #2 ultrasonography, #3 azoospermia, #4 ultrasound elastography, #5 apoptosis, and #6 epigenetics. **Figure 4E** highlights the top 25 keywords with the strongest citation bursts, with “assisted reproductive technology” having the longest burst and “sperm DNA fragmentation” the strongest at a score of 6.75. The latest keywords with strong citation bursts include “testicular sperm extraction, sperm DNA fragmentation, assisted reproductive technology, and outcome.” **Figure 4F** present the overlay visualizations of these keywords. The overlay visualization highlights recent keywords such as oxidative stress, activation, semen parameters, quality, nonobstructive azoospermia, sperm DNA fragmentation, *in vitro*, injection, diagnosis, growth, embolization, and microsurgery. These keywords, along with those showing strong citation bursts, suggest that current research on varicocele focuses on sperm quality, DNA fragmentation, clinical assessment, diagnosis, and management.

### 3.2 Real-world results

#### 3.2.1 Sperm analysis

**Figure 5:**
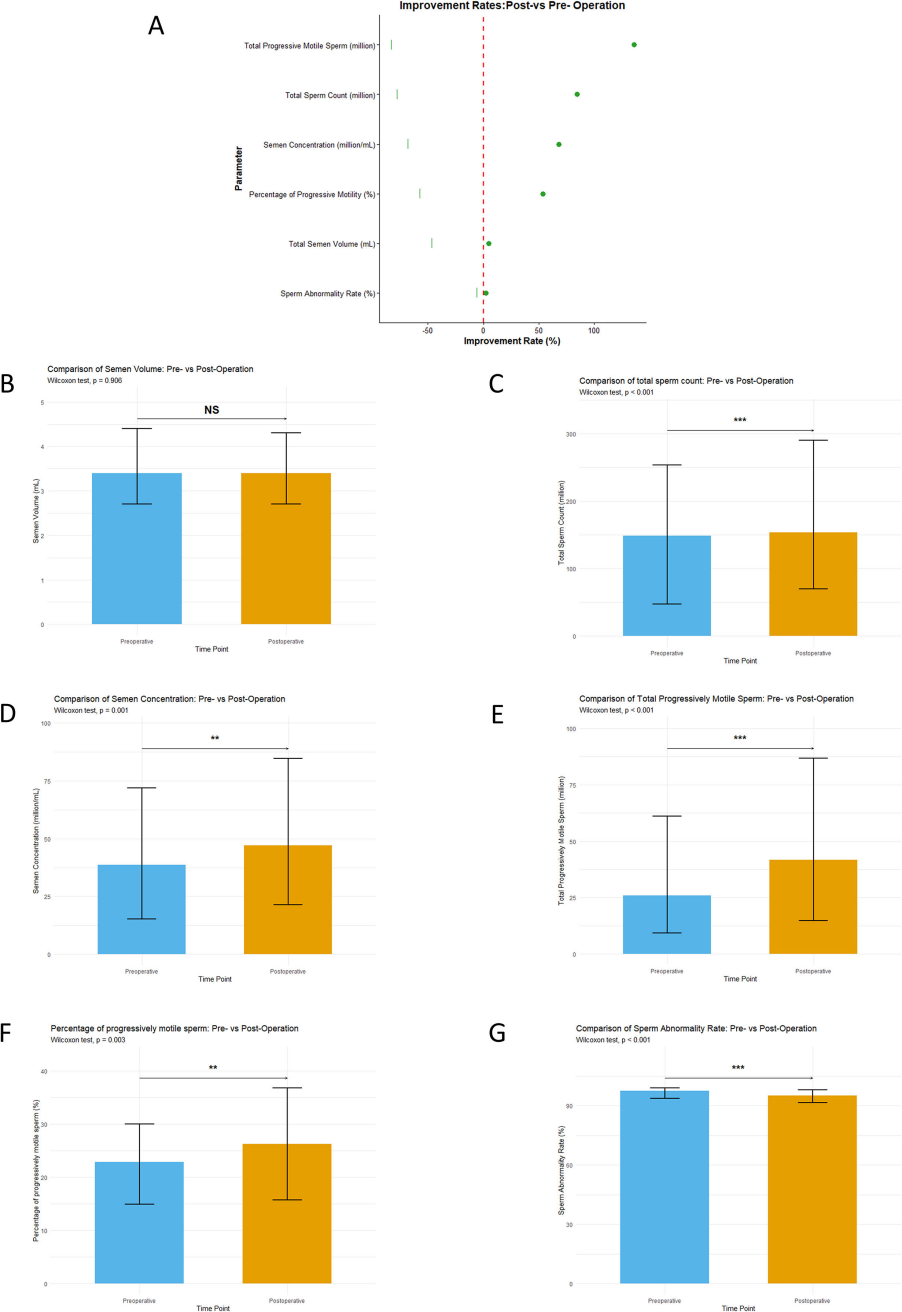
Compare Semen parameters before and after the operation. **(A)** The semen volume. **(B)** Total Sperm Count. **(C)** Semen Concentration. **(D)** Total Progressively Motile Sperm. **(E)** Percentage of progressively motile sperm. **(F)** Sperm Abnormality Rate. **(G)** The forest plot of the semen parameters improvement rate. (NS: p>0.05; *p< 0.05; **p< 0.01; ***p< 0.001).

A total of 113 patients met the inclusion criteria, with preoperative and postoperative data presented in **Table 3**, including semen parameters and Doppler B-ultrasound results (**Table 3**). The forest plot illustrates that following MSV, there is a 4.7% improvement in total semen volume, a 68.1% improvement in sperm concentration, an 84.2% improvement in the total number of sperm, a 53.7% improvement in the forward motility sperm rate, and a 135.7% improvement in the total progressively motile sperm count. Additionally, there is a 2.4% improvement in the sperm deformity rate (**Figure 5A**). Utilizing the Wilcoxon test, we observed significant improvements in total sperm count (**Figure 5C**), semen concentration (**Figure 5D**), total progressively motile sperm (**Figure 5E**), percentage of progressively motile sperm (**Figure 5F**), and sperm abnormality rate (**Figure 5G**) compared to preoperative values (P<0.05). However, the total semen volume did not show a statistically significant improvement compared to preoperative values (P = 0.906) (**Figure 5B**).

**Table 3:** The semen parameters and Doppler B-ultrasound result of preoperative and postoperative.

	Variable	Min	first_Qu.	Median	Mean	third_Qu.	Max
	Age	25.00	30.00	33.00	33.74	37.00	47.00
preoperative	Days of abstinence	2.00	3.00	4.00	4.25	5.00	7.00
	Semen volume	0.50	2.70	3.40	3.62	4.40	7.60
	Ph value	7.50	7.90	7.90	7.97	8.20	8.60
	Semen concentration	2.70	15.30	38.60	53.92	71.80	344.40
	Total sperm count	6.21	47.40	148.78	177.96	253.92	912.34
	Percentage of forward-moving sperm	0.00	14.90	22.90	23.55	30.00	65.30
	total number of forward motile sperm	0.00	9.26	25.96	48.29	61.09	624.36
	Sperm deformity rate	85.30	93.70	97.50	96.19	99.00	100.00
	Backflow time	2.00	5.00	8.00	7.81	10.00	16.00
	Inner diameter of the vein	2.10	2.68	3.20	3.11	3.50	5.30
postoperative	Days of abstinence	2.00	3.00	4.00	4.27	5.00	7.00
	Semen volume	1.00	2.70	3.40	3.61	4.30	10.20
	Ph value	7.00	7.90	7.90	7.93	7.90	8.60
	Semen concentration	3.80	21.40	47.00	63.17	84.60	405.10
	Total sperm count	12.16	69.72	153.66	218.04	290.40	1160.14
	Percentage of forward-moving sperm	0.00	15.70	26.30	27.52	36.80	66.10
	total number of forward motile sperm	0.00	14.91	40.82	60.35	86.48	358.75
	Sperm deformity rate	77.30	91.60	95.10	94.15	98.00	100.00

#### 3.2.2 Build models to analyze the factors influencing semen quality

##### 3.2.2.1 Build models to analyze the factors influencing the total progressively motile sperm

###### 3.2.2.1.1 Multiple linear regression model

**Figure 6:**
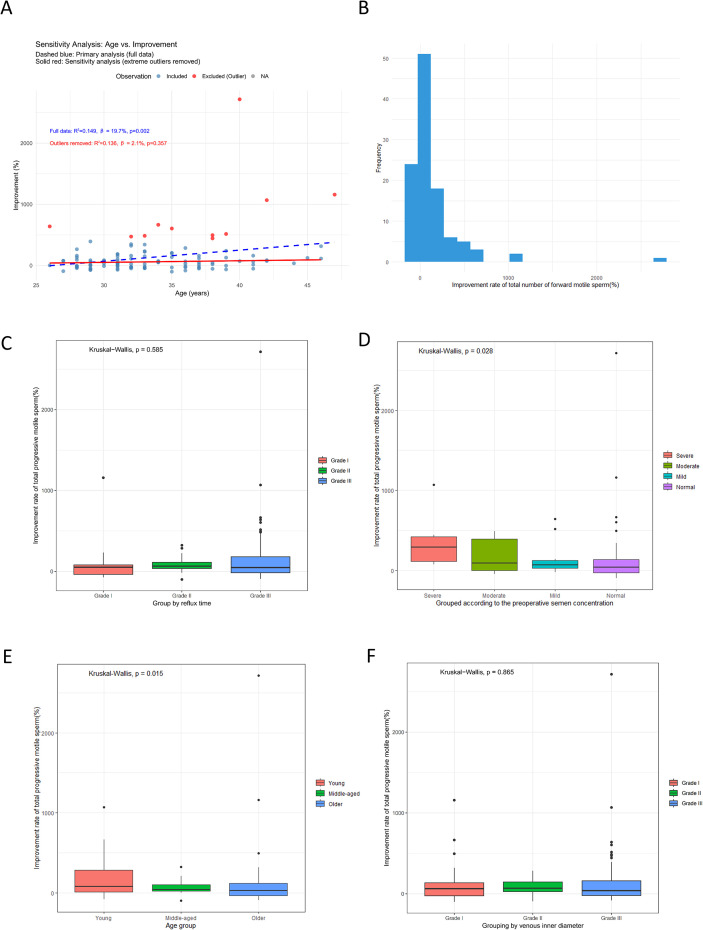
Predict the influencing factors of the improvement in total progressive motile sperm. **(A)** Sensitivity analysis of the association between age and the improvement. **(B)** Distribution map of Improvement. **(C)** The relationship between reflux time and the improvement of total progressive motile sperm. **(D)** The relationship between preoperative semen concentration and the improvement of total progressive motile sperm. **(E)** The relationship between age and the improvement of total progressive motile sperm. **(F)** The relationship between venous inner diameter and the improvement of total progressive motile sperm.

In constructing a multiple linear regression model, we incorporated variables such as age, pre-abstinence, pre-concentration, vein diameter, reflux time, type of surgery, and post-abstinence. Initial multicollinearity assessments revealed a substantial correlation between vein diameter and reflux time (r = 0.853), prompting the exclusion of “reflux time” from the model to mitigate multicollinearity issues. Subsequent evaluations confirmed the absence of multicollinearity and validated the model’s residuals (VIF < 5). The comprehensive multiple linear regression analysis performed on the complete dataset (n = 113) revealed that age is a significant positive predictor of enhanced forward motility in sperm following surgery (R² = 0.149, β = 19.700, p = 0.002) (**Table 4** and **Figure 6A**), but semen concentration (β = –35.800, p = 0.272), Vein Diameter (β = 19.100, p = 0.719), preoperative abstinence (β = –26.400, p = 0.219), postoperative abstinence (β = 11.100, p = 0.616), and surgical side (right: β = 618.000, p =0.075; left: β = 72.200, p = 0.311) showed no significant associations with improvement rate, as illustrated in **Table 4**. Conducted after the exclusion of extreme outliers of age in the sensitivity analysis (n = 102), the effect size was reduced and not statistically significant (R² = 0.136, β = 2.100, p = 0.357) (**Figure 6A**).

**Table 4:** Multiple linear regression model to analyze the influencing factors of total number of forward motile sperm.

Term	Estimate	Std.error	Statistic	Conf.low	Conf.high	P.value
Intercept	-580.000	304.000	-1.900	-1184.000	24.100	0.060
Age	19.700	6.360	3.100	7.130	32.300	0.002*
Pre-Abstinence	-26.400	21.400	-1.240	-68.800	15.900	0.219
Pre-Concentration	-35.800	32.400	-1.100	-100.00	28.500	0.272
Vein_Diameter	19.100	52.800	0.361	-85.600	124.000	0.719
Right-sided microsurgical high ligation	618.000	343.000	1.800	-62.800	1299.000	0.075
Left-sided microsurgical high ligation	72.200	70.900	1.020	-68.400	213.000	0.311
Post-Abstinence	11.100	22.100	0.504	-32.700	54.900	0.616

*P<0.05.

###### 3.2.2.1.2 Predict the influencing factors of the improvement in total progressive motile sperm

The distribution of the total progressively motile sperm improvement is shown in **Figure 6B**. To reconcile the findings from the multivariate model and univariate tests, the apparent discrepancy may arise because the non-linear relationships and interactions with other variables, which are not detected by the Kruskal-Wallis test. Subsequent Kruskal-Wallis tests revealed no significant differences in improvement rates when grouped by reflux time (p = 0.585, **Figure 6C**) or venous inner diameter (p = 0.865, **Figure 6F**). However, significant differences were observed when grouped by preoperative semen concentration (p = 0.028, **Figure 6D**) and age (p = 0.015, **Figure 6E**). The Kruskal-Wallis analyses confirmed that the improvement rate was significantly higher in the “severe oligozoospermia” group than in the “Normal” group (p = 0.020), and in the “Young” group compared to the “Older” group (p = 0.006).

##### 3.2.2.2 Build models to analyze the factors influencing the percentage of progressively motile sperm

###### 3.2.2.2.1 Multiple linear regression model

We developed a multiple linear regression model using age, pre-abstinence, pre-concentration, vein diameter, surgery type, and post-abstinence, ensuring no multicollinearity (VIF<5). However, none of the variables reached statistical significance (p > 0.05) as shown in **Table 5**. Preoperative semen concentration (β = 0.889, p = 0.495), age (β = 0.112, p = 0.661), preoperative abstinence (β = –0.239, p = 0.780), postoperative abstinence (β = 0.627, p = 0.478), and surgical side (right: β = 6.610, p = 0.634; left: β = 1.140, p = 0.690) showed no significant associations with improvement rate. Vein diameter had a marginally non-significant positive association (β = 3.720, p = 0.081).

**Table 5:** Multiple linear regression model to analyze the influencing factors of percentage of progressively motile sperm.

Term	Estimate	Std.error	Statistic	Conf.low	Conf.high	P.value
Intercept	-13.800	12.300	-1.130	-38.100	10.500	0.262
Age	0.112	0.255	0.440	-0.394	0.618	0.661
Pre-abstinence	-0.239	0.854	-0.280	-1.930	1.450	0.780
Pre-concentration	0.889	1.300	0.685	-1.690	3.460	0.495
Vein diameter	3.720	2.110	1.760	-0.470	7.920	0.081
Right-sided microsurgical high ligation	6.610	13.800	0.478	-20.800	34.000	0.634
Left-sided microsurgical high ligation	1.140	2.850	0.40	-4.510	6.790	0.690
Post-abstinence	0.627	0.879	0.713	-1.120	2.370	0.478

###### 3.2.2.2.2 Random forest model

**Figure 7:**
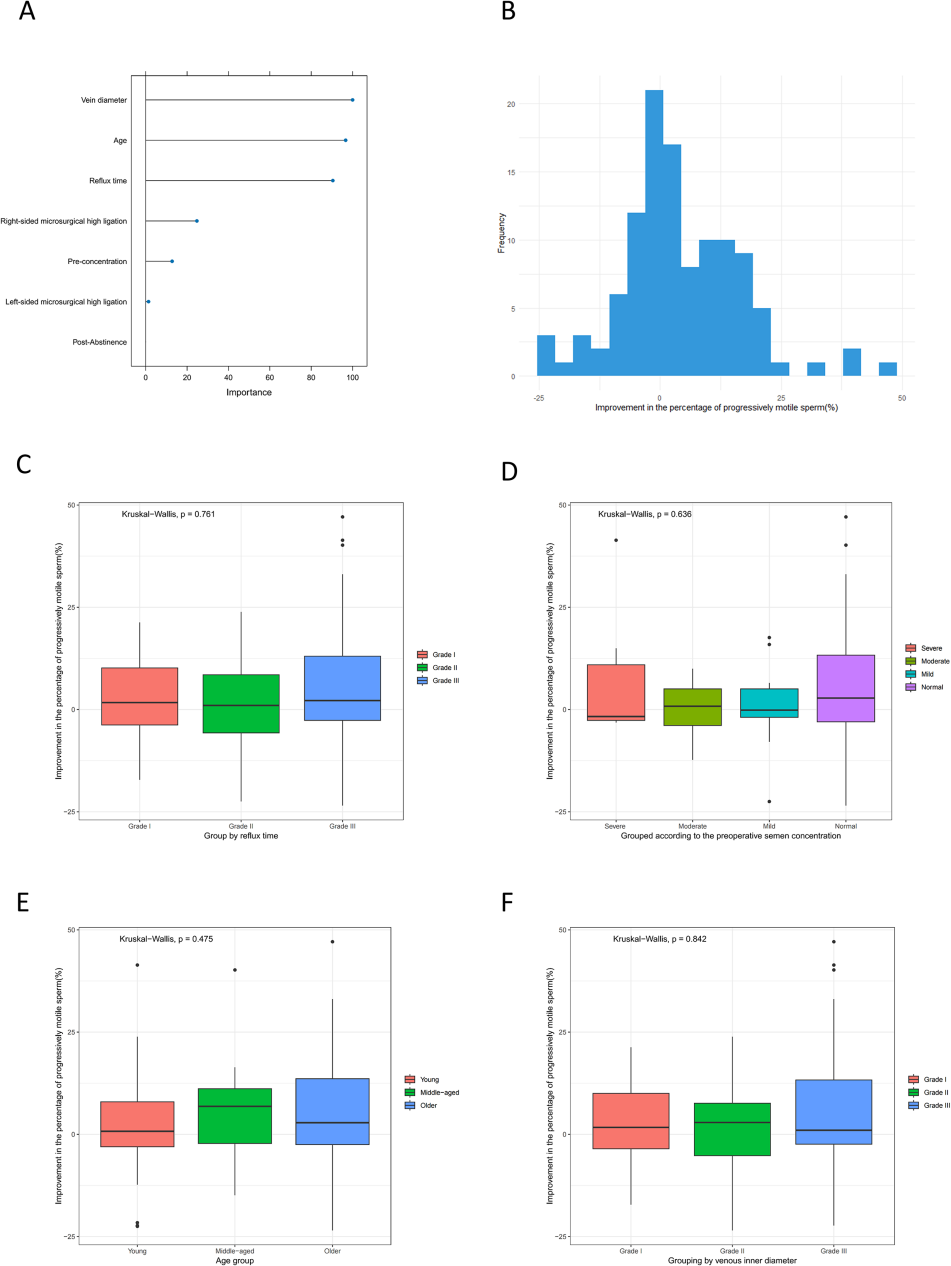
Predict the influencing factors of the improvement in the percentage of progressively motile sperm. **(A) **The random forest plot to determine the importance of influencing factors. **(B)** Distribution map of Improvement. **(C)** The relationship between reflux time and the improvement of the percentage of progressively motile sperm. **(D)** The relationship between preoperative semen concentration and the improvement of the percentage of progressively motile sperm. **(E)** The relationship between age and the improvement of the percentage of progressively motile sperm. **(F)** The relationship between venous inner diameter and the improvement of the percentage of progressively motile sperm.

Using a random forest model with factors like age, pre-abstinence, pre-concentration, vein diameter, reflux time, surgery type, and post-abstinence, we identified vein diameter as the key factor for enhancing the percentage of progressively motile sperm, followed by age and vein diameter again, as well as right-sided microsurgical high ligation (**Figure 7A**). **Figure 7B** illustrates the distribution of this improvement. The Kruskal-Wallis test was used to compare improvement rates across groups, revealing no significant differences when grouped by reflux time (p = 0.761, **Figure 7C**), preoperative semen concentration (p = 0.636, **Figure 7D**), age (p = 0.475, **Figure 7E**), or venous inner diameter (p = 0.842, **Figure 7F**).

##### 3.2.2.3 Build models to analyze the factors influencing of sperm abnormality rate

###### 3.2.2.3.1 Multiple linear regression model

To evaluate factors affecting sperm abnormality rates, we developed a multiple linear regression model using variables like age, abstinence duration, semen concentration, vein diameter, and surgery type. Despite passing multicollinearity tests (VIF<5), none of the variables were statistically significant (p > 0.05), as shown in **Table 6**. Each year of age increased improvement by 0.111 percentage points (β = 0.111, p = 0.226). A one-unit increase in sperm concentration led to a 0.522 percentage-point improvement (β = 0.522, p = 0.262), and a 1-mm increase in vein diameter resulted in a 0.811 percentage-point improvement (β = 0.811, p = 0.285). Other factors, such as preoperative and postoperative abstinence duration and surgical approach, also showed no significant impact. The model’s intercept was −4.210 (p = 0.338), which is not clinically meaningful.

**Table 6:** Multiple linear regression model to analyze the influencing factors of sperm abnormality rate.

Term	Estimate	Std.error	Statistic	Conf.low	Conf.high	P.value
Intercept	-4.210	4.370	-0.963	-12.90	4.460	0.338
Age	0.111	0.091	1.220	-0.070	0.291	0.226
Pre-abstinence	-0.021	0.305	-0.070	-0.625	0.583	0.944
Pre-concentration	0.522	0.463	1.13	-0.396	1.440	0.262
Vein_diameter	0.811	0.754	1.07	-0.685	2.310	0.285
Right-sided microsurgical high ligation	0.014	4.930	0.003	-9.770	9.800	0.998
Left-sided microsurgical high ligation	0.157	1.020	0.154	-1.860	2.170	0.878
Post-abstinence	-0.004	0.313	-0.014	-0.626	0.617	0.989

###### 3.2.2.3.2 Random forest model

**Figure 8:**
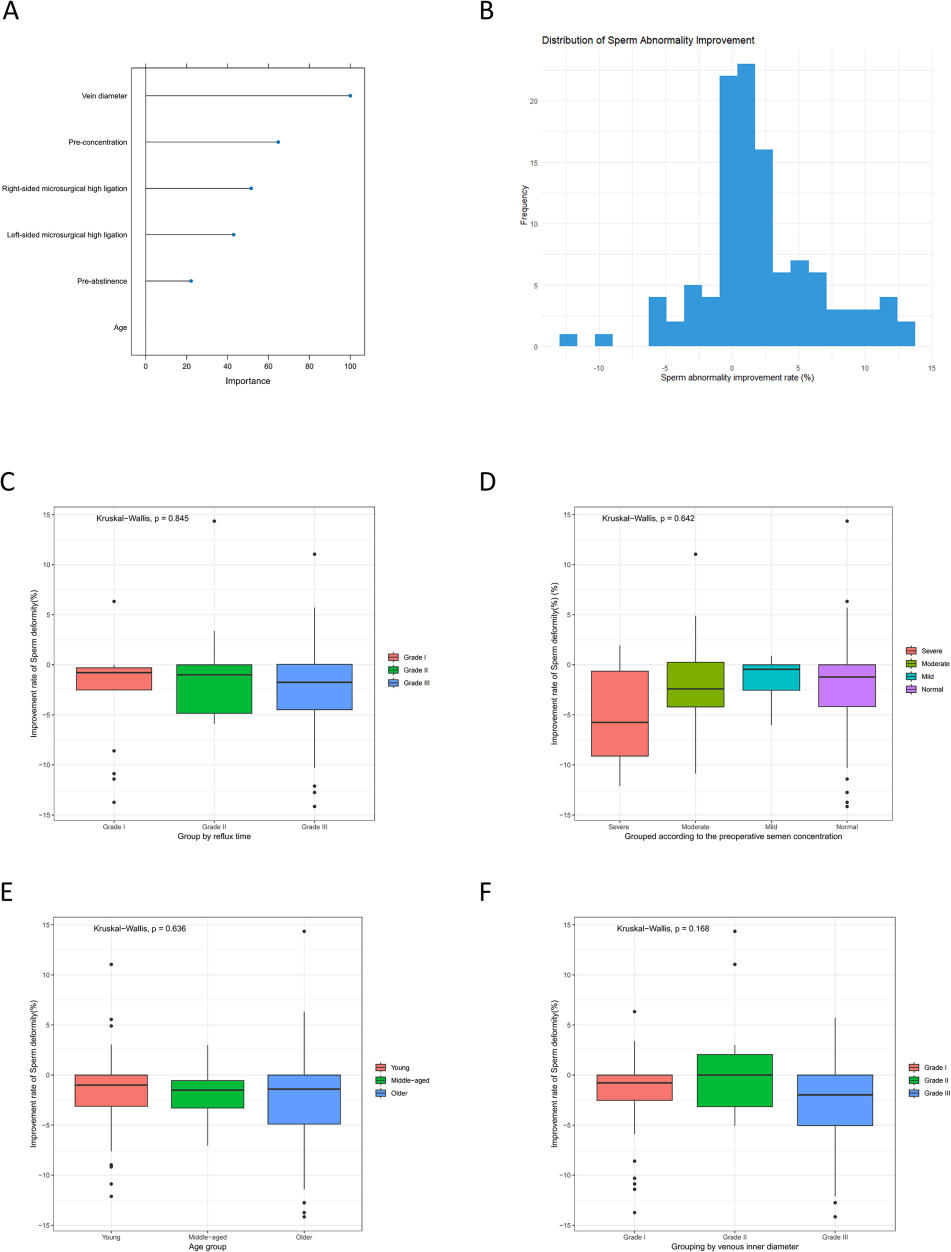
Predict the influencing factors of the improvement rate of sperm deformity. **(A)** The random forest plot to determine the importance of influencing factors. **(B) **Distribution map of improvement. **(C) **The relationship between reflux time and the improvement rate of sperm deformity. **(D)** The relationship between preoperative semen concentration and the improvement rate of sperm deformity. **(E)** The relationship between age and the improvement of the improvement rate of sperm deformity. **(F) **The relationship between venous inner diameter and the improvement rate of sperm deformity.

We employed a random forest approach to evaluate the contributions of age, pre- and post-abstinence durations, preoperative semen concentration, vein diameter, reflux time, and surgery type to the improvement rate of sperm abnormality. The analysis identified vein diameter as the strongest determinant, with preoperative semen concentration and right-sided microsurgical high ligation being the next most important variables (**Figure 8A**). **Figure 8B** presents the distribution of the improvement rate. Complementing the multivariate analysis, we conducted non-parametric group comparisons using the Kruskal-Wallis test. These tests indicated that the improvement rate did not differ significantly according to reflux time (p = 0.845, **Figure 8C**), preoperative semen concentration (p = 0.642, **Figure 8D**), age (p = 0.636, **Figure 8E**), or venous inner diameter (p = 0.168, **Figure 8F**).

## 4 Discussion

Integrating the bibliometric findings with our clinical data reveals alignment on several fronts. The keyword burst analysis identified ‘sperm DNA fragmentation’ and ‘oxidative stress’ as emerging frontiers; our clinical finding that venous diameter and reflux duration predict sperm quality improvements indirectly supports the mechanistic relevance of venous pressure-induced oxidative injury. Conversely, the bibliometric analysis highlighted ongoing debate regarding age as a predictor—our clinical data partially resolve this by demonstrating that while age shows a linear association in unadjusted analyses, stratification reveals that younger patients (<30 years) achieve the greatest benefit.

Bibliometric analyses of andrology literature over the past two to three decades reveal that varicocele represents one of the most extensively investigated topics within the discipline (19, 21). In our study, we included a total of 2,186 publications for bibliometric analysis, focusing on the contributions of various countries, journals, and authors to this area of research. Regarding publication trends, the annual output fluctuated between 70 and 129 publications from 2004 to 2014. However, over the past decade, there has been a steady increase in output, consistently exceeding 100 articles per year, which suggests a growing research interest in this topic. The United States, China, and Italy emerged as the three most prolific countries, with the United States also leading in total citations, indicating sustained engagement from researchers in major global scientific communities. Notably, author Agarwal A was identified as the most prolific contributor, having published the highest number of papers and receiving the greatest number of citations, thereby establishing himself as one of the most influential and actively involved researchers in this field. The journal *Andrologia* published the highest number of articles, followed by *Urology* and *The Journal of Urology*, highlighting the sustained research interest within the andrology community. Among the top ten co-cited references exhibiting the highest burst strength, all exceeding a value of 21, the most pronounced burst was identified in a study examining the impact of varicocelectomy on improving sperm parameters, including sperm count and motility (19). The paper with the second-highest burst strength concluded that microsurgical varicocelectomy (MSV) results in higher natural pregnancy rates and lower postoperative recurrence and hydrocele rates compared to conventional techniques (20). Conversely, another highly cited paper argued that varicocelectomy may not be an effective treatment for men with idiopathic oligoasthenospermia (22).

Over the past few decades, laparoscopic varicocelectomy has garnered significant attention; however, in the last ten years, it has largely been supplanted by microsurgical techniques. Surgical intervention for varicocele is endorsed by professional organizations, including the American Urological Association (AUA) and the American Society for Reproductive Medicine (ASRM) (11).

Our study indicates that following MSV, there were improvement rates of 68.1% in sperm concentration, 84.2% in total sperm count, 53.7% in the percentage of progressively motile sperm, and 135.7% in the total number of progressively motile sperm. MSV demonstrated efficacy in enhancing sperm concentration, progressive motility, and normal sperm morphology. Thus, it represents a valid and effective treatment modality for patients with isolated sperm defects, significantly rectifying their respective semen abnormalities and enhancing their prospects for natural conception (23).

The ten most frequently occurring keywords in the domain of varicocele research include: male infertility, male, oxidative stress, parameters, sperm, repair, fertility, infertility, ligation, and MSV. Analysis using CiteSpace’s timezone and overlay visualization tools reveals that oxidative stress, semen parameters, quality, sperm DNA fragmentation, and clinical evaluation are presently the focus of considerable scholarly interest.

In conclusion, we utilized comprehensive bibliometric analysis techniques to systematically examine the literature that has substantially influenced the current state and emerging trends in this field of research, thereby deriving the valuable insights previously discussed. Our findings indicate that the uneven geographical distribution of studies and fragmented author collaborations may partially account for the paucity of more thorough investigations in this domain. Despite varicocele being characterized as a vascular disorder, and notwithstanding the extensive literature endorsing the advantages of treatment—especially minimally invasive surgical procedures—significant controversy persists (24). Persistent questions remain concerning the substantiality of the benefits, the extent to which semen quality can be genuinely enhanced, and whether such enhancements lead to significant improvements in pregnancy outcomes (25). Current studies do not provide sufficiently compelling evidence to resolve these uncertainties. To address this gap, we are undertaking a real-world study utilizing our surgical data to closely analyze the degree of improvement in semen parameters, aiming to identify more convincing evidence through specific indicators.

We conducted an analysis of semen quality in patients pre- and post-surgery and observed a significant enhancement in sperm concentration, total sperm count, percentage of progressively motile sperm, and the total number of progressively motile sperm. Additionally, there was a significant reduction in the rate of sperm abnormalities.

Previous research has demonstrated that younger patients with varicocele exhibit the most significant enhancement in the total percentage of progressively motile sperm following surgical intervention (17, 26). However, our study uncovered a noteworthy phenomenon: the data suggest that the improvement in the total percentage of progressively motile sperm post-surgery becomes more pronounced with increasing patient age (**Figure 6A**).

When stratified by age group, patients under 30 years demonstrated the most favorable surgical outcomes, evidenced by the highest increase in the percentage of progressively motile sperm. This was followed by the 30–40 years age group, while patients over 40 showed comparatively less improvement in sperm parameters post-surgery (**Figure 6F**). The random forest analysis further identified reflux duration, preoperative sperm concentration, and age as the three most significant influencing factors. Concerning male age, there is ongoing debate about the efficacy of microsurgical varicocelectomy (MSV) in older men. This controversy stems from the hypothesis that long-standing varicocele may cause irreversible testicular damage or that the testes in older men have a diminished capacity to recover from such damage (27).

The clinical implication is that if microsurgical varicocelectomy (MSV) proves to be less effective in older male patients, it may not be advisable to recommend this procedure for them. Instead, these individuals could be guided towards assisted reproductive technologies (ART). Nonetheless, certain studies (28, 29) have indicated that age should not be regarded as an absolute exclusion criterion for varicocele treatment. Indeed, our scatter plot reveals that, for some older patients, there was a more pronounced postoperative improvement in the percentage of progressively motile sperm, although this finding is not universally applicable.

Firat and Erdemir (28), in their assessment of varicocele treatment outcomes among couples of varying ages, observed enhancements in semen parameters across all age groups. While the post-surgical pregnancy rate was elevated in the younger male cohort compared to their older counterparts, this disparity did not reach statistical significance. Consequently, couples with a male partner over the age of 35 still possess a substantial likelihood of achieving natural conception following microsurgical varicocelectomy (MSV). It is important to note, however, that the age of the female partner remains the critical determinant for natural conception.

In a study investigating the predictive factors for achieving pregnancy following varicocele repair, Matkov et al. determined that men with a postoperative total motile sperm count exceeding 20 million were more likely to successfully conceive through less invasive assisted reproductive technologies, such as natural conception or intrauterine insemination, as opposed to utilizing more complex methods like *in vitro* fertilization (30).

Pre-IVF/ICSI clinical microsurgical varicocelectomy (MSV) in individuals with oligospermia or non-obstructive azoospermia has been correlated with discernible clinical advantages. For couples utilizing assisted reproductive technology (ART) to attain pregnancy, varicocele repair may enhance semen parameters and potentially decrease the number of ART cycles necessary for achieving a successful pregnancy (31).

Men with infertility who undergo microsurgical varicocelectomy (MSV) are frequently considered candidates for less invasive and more cost-effective assisted reproductive technologies (ART). Furthermore, for those who still necessitate intracytoplasmic sperm injection (ICSI) following varicocele repair, both pregnancy and live birth rates are enhanced compared to individuals who undergo ICSI without prior varicocele repair (32, 33). While our study corroborates that MSV can enhance sperm quality, our primary concern remains its subsequent effect on pregnancy and live birth rates.

A prolonged duration of preoperative venous reflux and increased reflux severity are correlated with a heightened risk of diminished postoperative progressive sperm motility and decreased sperm concentration, thereby emphasizing the significance of reflux duration as a predictor of sperm quality (34). The resolution of left-sided varicocele reflux, as observed via color Doppler ultrasound, serves as an independent predictor of partner pregnancy following varicocele surgery (35, 36). Our study revealed that with an increase in reflux duration, there was a more pronounced improvement in the percentage of postoperative semen progressive motility, although this difference did not reach statistical significance, likely due to the limited sample size. This observation aligns with findings reported by other researchers.

Our random forest model identified venous diameter, age, and reflux duration as the three most significant predictors of enhancement in the percentage of progressively motile sperm post-surgery. Conversely, the primary predictors of improvement in the rate of sperm deformity were venous diameter, preoperative sperm concentration, and right-sided surgery. Furthermore, a meta-analysis indicated that a larger venous diameter and higher preoperative sperm concentration are correlated with more substantial improvements in semen quality following surgical intervention (37).

Clinicians and researchers frequently utilize the diameter of the spermatic vein as the principal criterion for assessing varicocele severity. However, recent studies have increasingly scrutinized the effectiveness of surgical interventions for varicocele. It is imperative to address more foundational concerns, particularly the adequacy of relying solely on venous diameter as a grading standard. Our findings suggest that a more refined classification system could be established by incorporating multiple factors, including venous diameter, patient age, and reflux duration, to enhance clinical decision-making.

Several limitations should be acknowledged. First, while our study suggests significant improvements in semen parameters following MSV, we acknowledge the absence of pregnancy and live birth outcomes as a major limitation. Semen parameters are surrogate endpoints; the ultimate clinical goal is successful conception and live birth. Future studies should incorporate longitudinal follow-up to capture these critical endpoints. Second, this was a single-center, retrospective case series without a control group (e.g., untreated varicocele patients or those undergoing alternative treatments). Third, causal inferences regarding the effectiveness of MSV are constrained; we can only report associations and within-subject improvements. Fourth, the sample size (n=113) is modest, which may limit statistical power for detecting small effect sizes and precludes robust subgroup analyses. Fifth, the lack of an external validation cohort means our predictive models (regression and random forest models) require confirmation in independent datasets. Therefore, our findings should be considered hypothesis-generating rather than definitive, and they underscore the need for multicenter prospective studies with larger sample sizes and control arms.5 Conclusion.

Varicocele research increasingly focuses on oxidative stress and sperm DNA fragmentation. Microsurgical varicocelectomy significantly improves semen parameters, with preoperative concentration, age, and reflux duration as potential predictors—though larger prospective validation is needed. It is important to note that while bibliometric analysis highlights ‘microsurgical varicocelectomy’ as a high-frequency keyword, this reflects research activity rather than an endorsement of universal clinical application. Current guidelines recommend careful patient selection, and surgery is not routinely advised for all men with varicocele, especially those with mild abnormalities or normal semen parameters. Refined grading remains preliminary and should not guide clinical practice. Future multicenter studies with long-term pregnancy outcomes are required to establish robust selection criteria.

## Data Availability

The raw data supporting the conclusions of this article will be made available by the authors, without undue reservation.
